# CeO_2_-Modified Ni_2_P/Fe_2_P as Efficient Bifunctional Electrocatalyst for Water Splitting

**DOI:** 10.3390/ma18102221

**Published:** 2025-05-11

**Authors:** Xinyang Wu, Dandan Wang, Yongpeng Ren, Haiwen Zhang, Shengyu Yin, Ming Yan, Yaru Li, Shizhong Wei

**Affiliations:** 1 School of Materials Science and Engineering, Henan University of Science and Technology, Luoyang 471000, China; 13243386137@163.com (X.W.); 13343791260@163.com (D.W.); ren_yp123@163.com (Y.R.); 2 Henan Key Laboratory of High-Temperature Metal Structural and Functional Materials, National Joint Engineering Research Center for Abrasion Control and Molding of Metal Materials, Henan University of Science and Technology, Luoyang 471000, China; 3 Longmen Laboratory, Luoyang 471000, China; 4 Longbai Group Co., Ltd., Jiaozuo 454191, China; zhanghaiwen@lomonbillions.com (H.Z.); yinshengyu@lomonbillions.com (S.Y.); ym@lomonbillions.com (M.Y.)

**Keywords:** transition metal phosphide, layered double hydroxide, cerium dioxide, OER, HER

## Abstract

Developing efficient bifunctional electrocatalysts with excellent stability at high current densities for overall water splitting is a challenging yet essential objective. However, transition metal phosphides encounter issues such as poor dispersibility, low specific surface area, and limited electronic conductivity, which hinder the achievement of satisfactory performance. Therefore, this study presents the highly efficient bifunctional electrocatalyst of CeO_2_-modified NiFe phosphide on nickel foam (CeO_2_/Ni_2_P/Fe_2_P/NF). Ni_2_P/Fe_2_P coupled with CeO_2_ was deposited on nickel foam through hydrothermal synthesis and sequential calcination processes. The electrocatalytic performance of the catalyst was evaluated in an alkaline solution, and it exhibited an HER overpotential of 87 mV at the current density of 10 mA cm^−2^ and an OER overpotential of 228 mV at the current density of 150 mA cm^−2^. Furthermore, the catalyst demonstrated good stability, with a retention rate of 91.2% for the HER and 97.3% for the OER after 160 h of stability tests. The excellent electrochemical performance can be attributed to the following factors: (1) The interface between Ni_2_P/Fe_2_P and CeO_2_ facilitates electron transfer and reactant adsorption, thereby improving catalytic activity. (2) The three-dimensional porous structure of nickel foam provides an ideal substrate for the uniform distribution of Ni_2_P, Fe_2_P, and CeO_2_ nanoparticles, while its high conductivity facilitates electron transport. (3) The incorporation of larger Ce^3^⁺ ions in place of smaller Fe^3^⁺ ions leads to lattice distortion and an increase in defects within the NiFe-layered double hydroxide structure, significantly enhancing its catalytic performance. This research finding offers an effective strategy for the design and synthesis of low-cost, high-potential catalysts for water electrolysis.

## 1. Introduction

With the rapid depletion of fossil fuels and growing concerns about their negative environmental impact, there is an urgent need for renewable energy sources and storage technologies to replace fossil fuels [1,2,3]. Hydrogen, with its high storage density and environmental friendliness, has emerged as a promising green fuel to replace fossil fuels [4,5,6]. However, the kinetics of the hydrogen evolution reaction (HER) and oxygen evolution reaction (OER), which occur at the cathode and anode, respectively, are hindered by the multi-proton process and high energy barriers associated with electrolyzed water [7,8,9]. Currently, precious metals such as platinum (Pt) and oxides like iridium dioxide (IrO_2_) and ruthenium dioxide (RuO_2_) are the most effective electrocatalysts. However, their high cost and limited availability impede the large-scale and commercial application of water splitting. Therefore, there is a growing research focus on exploring cost-effective, stable, and environmentally friendly non-precious metal catalysts to replace traditional precious metal catalysts in electrolytic water technology [10,11,12,13].

In recent years, transition metal phosphides (TMPs) have emerged as highly promising bifunctional catalysts due to their structural similarity to hydrogenase, adjustable valence, diverse metal properties and phases, and ability to exhibit excellent catalytic activity in both the HER and the OER [14,15,16]. The incorporation of phosphorus in these catalysts enhances the release of hydrogen by promoting the weak ‘ligand effect’ of metal–phosphorus bonds [17,18,19]. For example, Tian et al. developed a novel ultrathin folded Ni_2_P/Fe_2_P nanosheet electrocatalyst that requires only a 237 mV overpotential for an OER at 10 mA cm⁻^2^ [20]. Yue et al. demonstrated the synthesis of Ni-Fe bimetallic phosphide nanocube clusters on NF substrates through simple two-step hydrothermal reactions and low-temperature phosphating treatment. These clusters exhibited excellent HER and OER performance in a 1 M KOH electrolyte. At a current density of 20 mA cm^−2^, the initial overpotentials were measured at 286 mV and 242 mV, with Tafel slopes of 69 mV dec^−1^ and 126 mV dec^−1^, respectively. The introduction of phosphorus accelerates mass transfer in the reaction [21]. The synergistic effect of bimetallic nickel-iron phosphide enhances the activity of both the HER and OER. Phosphorus-containing structures in these bimetallic phosphides exhibit excellent electronegativity, facilitating rapid electron transfer between the metal and phosphorus [22,23,24]. However, bimetallic transition metal phosphides exhibit limited capabilities in breaking down water molecules and face challenges in balancing the adsorption energies of hydrogen and oxygen intermediates, which constrains their performance in the OER and HER. Furthermore, there is a need to improve their conductivity for better performance.

Cerium dioxide (CeO_2_), a typical rare earth metal oxide, demonstrates a reversible exchange of surface oxygen ions, high oxygen storage capacity, and oxygen vacancies owing to the flexible conversion between Ce^4^⁺ and Ce^3^⁺ [25,26]. As a significant accelerator, CeO_2_ possesses excellent electronic and ionic conductivity, high oxygen storage capacity, and strong polarization ability [27,28,29]. Its use as an accelerator to improve electrocatalytic performance is widespread, thanks to its abundant oxygen vacancies and strong affinity for oxygen-containing substances. The combination of CeO_2_ with metals like nickel (Ni), nickel phosphide (Ni_2_P), and cobalt phosphide (CoP) has been shown to effectively enhance the catalytic performance of the HER by promoting hydrolysis and enhancing hydrogen adsorption under alkaline conditions [30,31]. Additionally, heterostructures based on CeO_2_, such as Ni(OH)_2_-CeO_2_, CeO_2_/CoS_2_, and CoP_3_/CeO_2_/C, exhibit excellent catalytic activity for the OER due to their high oxygen storage/release rate and strong electron interaction with other substrates, thus improving OER catalysis [32,33,34]. Moreover, CeO_2_ not only provides significant corrosion protection but also exhibits excellent mechanical resistance, thereby enhancing the durability of coupled electrocatalysts [35,36]. For instance, Huang et al. designed a highly efficient bifunctional electrocatalyst by modifying Fe-doped Ni_2_P nanosheets with CeO_2_ nanoparticles on a nickel foam substrate. The catalyst exhibits an HER overpotential of 292 mV at a current density of 1000 mA cm^−2^. At a current density of 500 mA cm^−2^, the OER overpotential is 240 mV. The catalyst exhibited good stability, with no significant decay after 120 h [37]. CeO_2_ demonstrates dual functionality by offering effective corrosion resistance while simultaneously providing robust mechanical properties, thereby substantially improving the operational lifespan of hybrid electrocatalytic systems. This oxide additive enables sustained catalytic performance under extreme environmental conditions without significant degradation. CeO_2_ modification represents a new approach to enhance the performance of OER electrocatalysts. Its multivalent properties enable fine-tuning of the electronic structure and OER performance of the catalytic matrix through electronic exchange [38]. Chen et al. studied a novel heterostructure, where metal–organic framework (MOF)-derived Co_0.4_Ni_1.6_P nanowire arrays were deposited on the surface of CeO_2_ nanoparticles. This interface engineering strategy created abundant oxygen vacancies and increased the number of electrocatalytic active sites, leading to excellent OER performance. The OER overpotentials were measured at 268 mV and 343 mV at 10 mA cm^−2^ and 100 mA cm^−2^ [39]. The CeO_2_-mediated Ce^4+^/Ce^3+^ redox process generated rich oxygen vacancies and excellent oxygen storage capacity, while the electronic interaction between CeO_2_ and the coupled electrocatalyst regulated the intermediate of interfacial electron transfer, adsorption, and activation energy, thereby improving OER kinetics [40].

Based on these considerations, this study introduced CeO_2_ onto two-dimensional Ni_2_P/Fe_2_P nanosheets based on an NF substrate to prepare a CeO_2_/Ni_2_P/Fe_2_P/NF nanocomposite as an efficient bifunctional catalyst for hydrogen and oxygen evolution. Notably, the catalyst required merely 87 mV overpotential to deliver 10 mA cm⁻^2^ for an HER, while achieving 150 mA cm⁻^2^ OER current density at a 228 mV overpotential. After a continuous 200 h cycle test, the stability of the HER and OER remained at 91.2% and 97.3%, respectively, without significant attenuation. The Tafel slopes were 97.32 mV dec^−1^ and 119.68 mV dec^−1^ for the HER and OER, respectively, indicating good catalytic activity. The introduction of CeO_2_ altered the morphology and electronic structure of Ni_2_P/Fe_2_P/NF, significantly enhancing charge transport during the catalytic process. Additionally, CeO_2_ provided corrosion protection and showed excellent mechanical resistance, thereby improving the durability of the coupled electrocatalyst.

## 2. Experiment

### 2.1. Chemicals and Reagents

The experimental reagents included cerium nitrate hexahydrate (Ce(NO_3_)_2_·6H_2_O), nickel nitrate hexahydrate (Ni(NO_3_)_2_·6H_2_O), iron nitrate nonahydrate (Fe(NO_3_)_3_·9H_2_O), ammonium fluoride (NH_4_F), urea (CO(NH_2_)_2_), sodium hypophosphite (NaH_2_PO_2_·H_2_O), potassium hydroxide (KOH), and nickel foam (NF). The water used in the experiment underwent treatment by an ultrapure purification system.

### 2.2. Nickel Foam Pretreatment

After being cut to 2 × 2 cm^2^ dimensions, the nickel foam was ultrasonically cleaned in acetone and 3 M hydrochloric acid (15 min) for surface activation. Subsequent purification included multiple rinses with deionized water and absolute ethanol, culminating in 12 h vacuum drying at 60 °C.

### 2.3. Preparation of CeO_2_/NiFe-LDH/NF Precursor

The CeO_2_/NiFe-LDH/NF precursor was prepared using a one-pot hydrothermal method. The synthesis began with the dissolution of metal nitrates (0.5452 g Ni(NO_3_)_2_·6H_2_O and 0.2525 g Fe(NO_3_)_3_·9H_2_O) along with 0.046 g NH_4_F and 0.375 g urea in 25 mL deionized water. Following 30 min of magnetic stirring at room temperature to achieve complete dissolution, the transparent solution and pretreated nickel foam substrate were placed together in a 50 mL Teflon-lined stainless-steel autoclave. The autoclave was sealed, and the reaction was carried out at 120 °C for 12 h in a blast drying oven. After cooling the autoclave to room temperature, the nickel foam was removed, washed with deionized water several times, and then vacuum-dried overnight to obtain the CeO_2_/NiFe-LDH/NF precursor.

### 2.4. Preparation of CeO_2_/Ni_2_P/Fe_2_P/NF Catalyst

The preparation of the CeO_2_/Ni_2_P/Fe_2_P/NF catalyst involves two calcination steps based on the synthetic precursor. In the first step, the CeO_2_/NiFe-LDH/NF precursor was calcined in an argon-filled tube furnace at 450 °C for 2 h. Afterward, 0.3 g of NaH_2_PO_2_·H_2_O was weighed and placed upstream of the tube furnace, while the precursor from the first step of calcination was placed downstream. The sample was then heated to 350 °C at a rate of 5 °C per minute in an argon atmosphere and maintained at this temperature for two hours. The resulting product was named CeO_2_/Ni_2_P/Fe_2_P/NF. Depending on the different amounts of Ce(NO_3_)_3_·6H_2_O (0.0054 g, 0.0163 g, 0.0271 g, 0.0380 g, and 0.0543 g), the products were labeled as CeO_2_/Ni_2_P/Fe_2_P/NF-1, CeO_2_/Ni_2_P/Fe_2_P/NF-2, CeO_2_/Ni_2_P/Fe_2_P/NF-3, CeO_2_/Ni_2_P/Fe_2_P/NF-4, and CeO_2_/Ni_2_P/Fe_2_P/NF-5, respectively. The preparation process for Ni_2_P/Fe_2_P/NF is similar to that of CeO_2_/Ni_2_P/Fe_2_P/NF, except that Ce(NO_3_)_3_·6H_2_O is not introduced.

### 2.5. Characterization

Phase identification was performed by X-ray diffraction (XRD) measurements using a Bruker X diffractometer (Bruker AXS, Karlsruhe, Germany), while chemical state analysis was carried out via X-ray photoelectron spectroscopy (XPS) with an ESCALAB 250 Xi system (Thermo Fisher Scientific, East Grinstead, UK) to determine surface composition and valence states. The morphology of the samples was analyzed using both field emission scanning electron microscopy (SEM) on a JSM-IT800 instrument (JEOL Ltd., Tokyo, Japan) and transmission electron microscopy (TEM) on an FEI Talos F200 S instrument (Thermo Fisher Scientific, Hillsboro, OR, USA).

### 2.6. Electrochemical Measurement

Electrochemical characterizations were performed with a standard three-electrode configuration on a Princeton workstation, employing Ag/AgCl as the reference, graphite as the counter electrode, and nickel foam-supported catalyst as the working electrode. Noble metal standard electrodes were obtained by ethanol dispersion (1 mL) of weighed Pt/C or RuO_2_ samples and subsequent coating on nickel foam. The mixture was then subjected to ultrasonication for 10 min. Subsequently, 50 μL of Nafion solution (5 wt%) was added to the mixture and ultrasonicated for an additional 25 min to obtain a uniform black ink. These inks were evenly dropped onto the nickel foam and dried using a digital infrared baking lamp. All electrochemical tests were performed in a 1.0 M KOH solution with a pH of 13.7. All data presented in this paper were converted to relative values with respect to the reversible hydrogen electrode, following the formula E_RHE_ = E_Ag/AgCl_ + 0.197 + 0.0592 × pH − iR [41]. Linear sweep voltammetry (LSV) curves for the OER and HER were recorded in 1 M KOH at a scan rate of 5 mV s^−1^. The Tafel diagram was obtained using the equation η = a + blog(j) [42]. The electrochemical active surface area was quantified by analyzing capacitive currents in non-faradaic CV scans (60–100 mV s⁻^1^ scan rates), where the double-layer capacitance (Cdl) was determined via linear fitting. Additional characterization included impedance spectroscopy (100 kHz–0.01 Hz, 10 mV amplitude) and stability assessment through constant-potential chronoamperometry.

## 3. Results and Discussion

### 3.1. Structure and Morphology Characterization

**Scheme 1:**
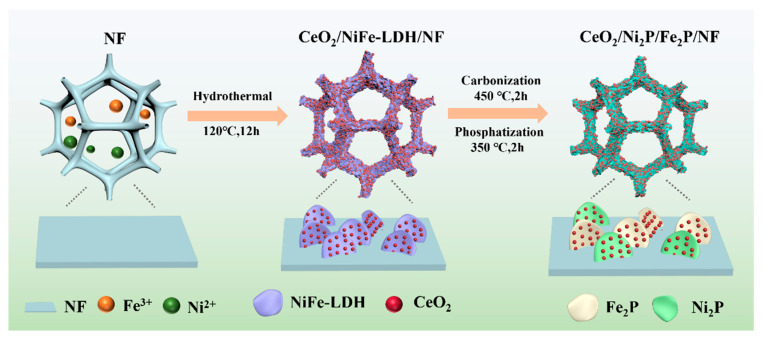
Illustration of the synthesis of CeO_2_/Ni_2_P/Fe_2_P/NF.

The preparation process of CeO_2_/Ni_2_P/Fe_2_P/NF composites is illustrated in Scheme 1. Initially, the CeO_2_/NiFe-LDH/NF precursor was synthesized through a hydrothermal method. As shown in Figure S1, the characteristic XRD reflection peaks, observed at 11.4°, 22.9°, 33.5°, 34.4°,39.0°, 59.9°, and 61.2°, correspond to the standard peaks found on the hydrotalcite standard card PDF#40-0215, confirming the successful synthesis of CeO_2_/NiFe-LDH/NF. Subsequently, the CeO_2_/Ni_2_P/Fe_2_P/NF composite was successfully obtained through a two-step calcination process.

**Figure 1:**
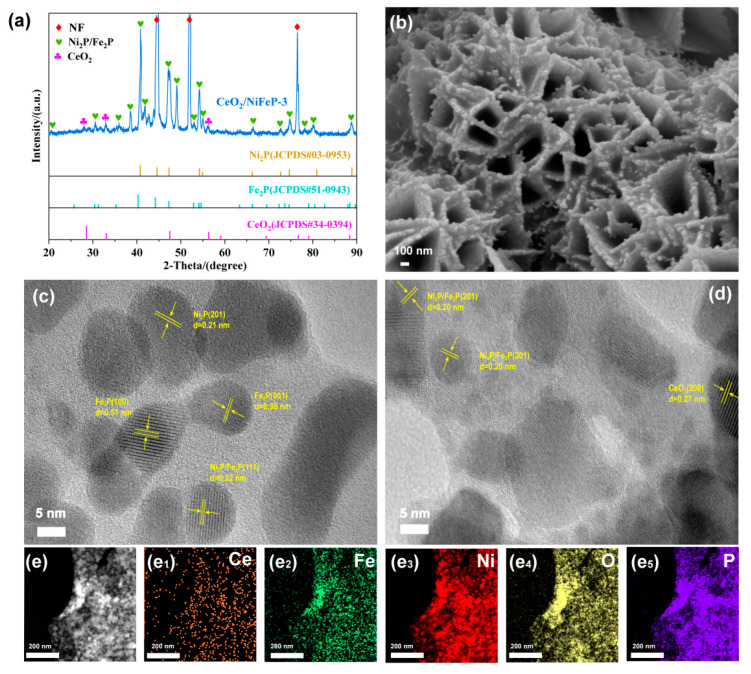
(**a**) XRD pattern and (**b**) SEM image of CeO_2_/Ni_2_P/Fe_2_P/NF-3, (**c**) HRTEM image of Ni_2_P/Fe_2_P/NF, (**d**) HRTEM image of CeO_2_/Ni_2_P/Fe_2_P/NF-3, and (**e**) EDS diagram of CeO_2_/Ni_2_P/Fe_2_P/NF-3, (**e1**–**e5**) distribution diagram of element.

As presented in Figure S2, the diagram reveals characteristic peaks from the nickel foam and phosphide. These phosphide peaks correspond to Ni_2_P (JCPDS#No. 03-0953) and Fe_2_P (JCPDS#No.51-0943), indicating the successful synthesis of Ni_2_P/Fe_2_P/NF. However, the XRD pattern does not exhibit a prominent peak for CeO_2_, which is likely due to the small size, low crystallinity, and low content of CeO_2_ nanoparticles. However, after partially amplifying the XRD pattern, a weak peak of CeO_2_ becomes visible (Figure 1a). The XRD pattern reveals distinct diffraction features characteristic of the synthesized phosphides. For Ni_2_P, the observed reflections at 2θ values of 40.8°, 44.6°, and 47.3° index to the (111), (201), and (210) crystallographic planes, respectively (JCPDS No. 03-0953), verifying phase purity. Parallel analysis of Fe_2_P demonstrates corresponding peaks at 40.3°, 44.3°, and 47.3° matching its standard (111), (201), and (210) lattice planes (JCPDS No. 51-0943), confirming the successful preparation of both target materials. The XRD analysis further revealed characteristic diffraction peaks at 2θ = 28.2°, 33.0°, 47.5°, and 56.3°, which were indexed to the (111), (200), (220), and (311) crystallographic planes of cubic CeO_2_ (JCPDS 34-0394). These distinctive reflections provide conclusive evidence for the successful integration of cerium oxide into the composite material. This confirms the successful preparation of CeO_2_/Ni_2_P/Fe_2_P/NF.

The morphology of the samples was analyzed using SEM, as illustrated in Figure 1b. The CeO_2_/Ni_2_P/Fe_2_P/NF structure retains its nanosheet form post-phosphating. Figure S3 depicts the changes in material morphology of CeO_2_/Ni_2_P/Fe_2_P/NF and Ni_2_P/Fe_2_P/NF during hydrothermal, calcination, and phosphating processes. These nanosheets are interconnected and stacked, forming an open porous structure that facilitates the exposure of abundant active sites and enhances mass transfer. Additionally, the smooth Ni_2_P/Fe_2_P/NF nanosheet arrays are densely and uniformly distributed on the nickel foam. In contrast, the surface of the CeO_2_/Ni_2_P/Fe_2_P/NF nanosheet appears rough due to the presence of some particles. The element distribution and its proportion are shown in Figure S4 and Table S1. The nickel foam substrate, with its excellent electrical conductivity and mechanical strength, particularly its three-dimensional (3D) open macroporous channels, promotes the effective diffusion of electrolytes and gases generated during electrolysis.

HRTEM was performed to gain a deeper understanding of the crystal structure of materials. Figure 1c displays the HRTEM images of Ni_2_P/Fe_2_P/NF, revealing the crystal plane spacing of 0.35 nm and 0.51 nm, corresponding to the (001) and (100) crystal planes of Fe_2_P, respectively. The 0.21 nm lattice spacing originates from the (201) crystal plane of Ni_2_P, while the 0.22 nm lattice spacing is attributed to the (111) crystal plane of both Fe_2_P and Ni_2_P, confirming the presence of Ni_2_P/Fe_2_P/NF in the catalyst. Figure 1d displays the HRTEM image of CeO_2_/Ni_2_P/Fe_2_P/NF-3, where the 0.27 nm lattice spacing corresponds to the (200) crystal plane of CeO_2_. Additionally, the lattice spacings of 0.22 nm and 0.20 nm correspond to the (111) and (201) crystal planes, respectively, thereby confirming the presence of Fe_2_P and Ni_2_P. The results from XRD, SEM, and TEM collectively confirm the successful preparation of CeO_2_/Ni_2_P/Fe_2_P/NF structure materials.

**Figure 2:**
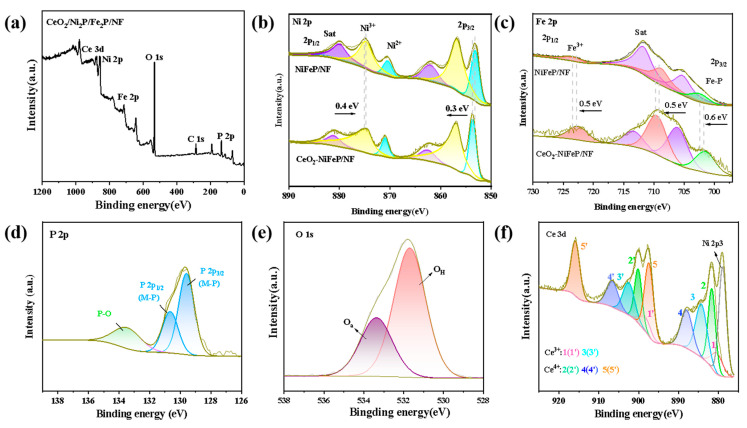
XPS spectra of CeO_2_/Ni_2_P/Fe_2_P/NF-3: (**a**) a full scan survey, (**b**) Ni 2p, (**c**) Fe 2p, (**d**) P 2p, (**e**) O 1s, and (**f**) Ce 3d.

The surface elemental composition and chemical state of the prepared CeO_2_/Ni_2_P/Fe_2_P/NF-3 electrode were further investigated by XPS. The full spectrum in Figure 2a reveals the presence of Ni, Fe, P, O, and Ce elements. The XPS spectra of Ni 2p in Figure 2b exhibits peaks at 874.5 eV and 856.8 eV, corresponding to 2p_1/2_ and 2p_3/2_ of Ni^3+^, respectively, while the peaks at 871 eV and 853.6 eV are attributed to 2p_1/2_ and 2p_3/2_ of Ni^2+^ (from Ni-P bond), accompanied by satellite peaks at 881.1 eV and 862.5 eV [43]. In Figure 2c, the XPS spectrum of Fe 2p reveals two spin-orbit peaks (Fe 2p_3/2_ and Fe 2p_1/2_) at 713.4 eV and 724.9 eV, respectively, accompanied by two satellite peaks at 710.4 eV and 716.8 eV. In addition, a peak was observed at 706.4 eV, indicating the presence of Fe-P bonds, confirming the formation of Fe_2_P [44]. This variation demonstrates that CeO_2_ effectively modulates the electronic structure of the Fe_2_P/Ni_2_P/CeO_2_ composite, generating favorable electronic configurations that synergistically enhance both HER and OER catalytic processes. The XPS spectrum of the P 2p region shown in Figure 2d exhibits signals at 129.6 eV and 130.6 eV, which correspond to P 2p_3/2_ and P 2p_1/2_, respectively. These signals are indicative of the metal–phosphorus (Ni-P or Fe-P) bond. The peak at 133.4 eV indicates the presence of a P-O bond due to surface oxidation of the catalyst [45]. In the O 1s spectrum shown in Figure 2e, the peak at 531.6 eV is attributed to the signal generated by the P-O bond or the hydroxyl group adsorbed on the oxide, while the peak at 533.3 eV corresponds to adsorbed oxygen [46]. The Ce 3d XPS spectrum presented in Figure 2f exhibits complex multiplet splitting characteristics. Prior to 880 eV, the observed features are attributed to Ni species. The characteristic doublet at binding energies of 898.3 eV and 902.5 eV corresponds to the Ce^3^⁺ oxidation state, while the triplet at 900.1 eV, 906.6 eV, and 915.8 eV is indicative of Ce^4^⁺ species. This mixed valence state suggests the coexistence of both cerium oxidation states in the material [47]. Figure S5 illustrates the XPS results for Ni_2_P/Fe_2_P/NF, revealing the surface elemental composition and chemical state of the electrode without Ce addition. The redox effect of Ce^4+^/Ce^3+^ contributes to abundant oxygen vacancies and excellent oxygen storage capacity. The electronic interaction between CeO_2_ and Ni_2_P/Fe_2_P can also adjust the CeO_2_ adsorption and activation energy, thereby enhancing the kinetics of the OER [48,49,50].

### 3.2. Electrochemical HER Performance

**Figure 3:**
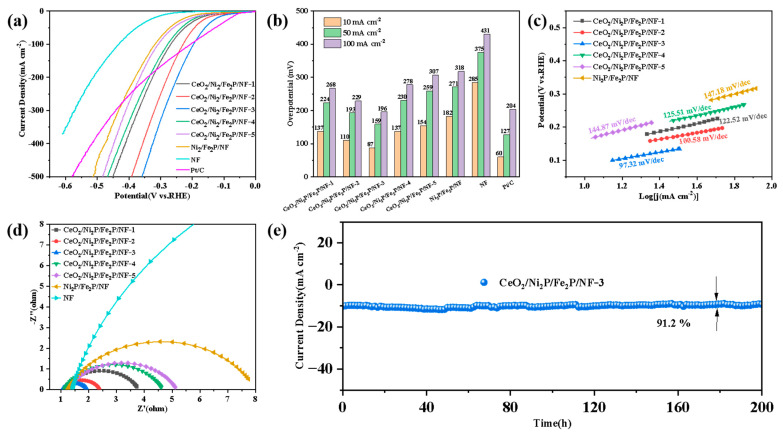
HER electrocatalytic performance in 1.0 M KOH: (**a**) LSV curve, (**b**) overpotential, (**c**) Tafel slope, (**d**) EIS spectrum, and (**e**) durability of CeO_2_/Ni_2_P/Fe_2_P/NF−3 determined by chronoamperometry at 10 mA cm^−2^.

The HER activity of the CeO_2_/Ni_2_P/Fe_2_P/NF-1 to CeO_2_/Ni_2_P/Fe_2_P/NF-5 electrocatalysts was evaluated in an alkaline solution (1.0 M KOH) using LSV linear sweep voltammetry at a scan rate of 5 mV s^−1^, in order to determine the optimal Ce content. The electrocatalytic activity of Ni_2_P/Fe_2_P/NF, NF, and commercial Pt/C (20 wt%) was also tested under the same conditions. As illustrated in Figure 3a,b, CeO_2_/Ni_2_P/Fe_2_P/NF-3 exhibits the lowest overpotential of 87 mV at 10 mA cm^−2^, which is lower than the overpotentials of CeO_2_/Ni_2_P/Fe_2_P/NF-1 (137 mV), CeO_2_/Ni_2_P/Fe_2_P/NF-2 (110 mV), CeO_2_/Ni_2_P/Fe_2_P/NF-4 (137 mV), and CeO_2_/Ni_2_P/Fe_2_P/NF-5 (154 mV). Additionally, the Ni_2_P/Fe_2_P/NF and NF electrocatalysts require larger overpotentials of 182 mV and 285 mV, respectively. These results indicate that the introduction of CeO_2_ significantly enhances the HER activity. It is important to note that Pt/C exhibits the highest activity at low current densities. However, once a certain overpotential is surpassed, CeO_2_/Ni_2_P/Fe_2_P/NF-3 demonstrates a greater current density than commercial Pt/C. This indicates that CeO_2_/Ni_2_P/Fe_2_P/NF-3 is more suitable for high current density operations, a crucial consideration for assessing the practical applications of the HER. The high HER activity of the catalyst can be attributed to the incorporation of CeO_2_, which is beneficial for improving the water decomposition activity of the catalyst [51,52].

To investigate the electrocatalytic kinetics of samples, the Tafel slope was determined by fitting the respective LSV curves. Figure 3c illustrates that the Tafel slope of the CeO_2_/Ni_2_P/Fe_2_P/NF-3 electrode is 97.32 mV dec⁻^1^, the lowest among all tested electrodes. In comparison, the Tafel slopes for CeO_2_/Ni_2_P/Fe_2_P/NF-1, CeO_2_/Ni_2_P/Fe_2_P/NF-2, CeO_2_/Ni_2_P/Fe_2_P/NF-4, CeO_2_/Ni_2_P/Fe_2_P/NF-5, Ni_2_P/Fe_2_P/NF, and NF electrodes are 122.52, 100.58, 125.51, 144.87, and 147.18 mV dec⁻^1^, respectively. This indicates that the incorporation of CeO_2_ promotes the dissociation of H_2_O and accelerates the recombination of hydrogen, confirming that the CeO_2_/Ni_2_P/Fe_2_P/NF-3 electrode exhibits faster HER kinetics [53].

Additionally, EIS was employed to investigate the electrochemical behavior and conductivity of the samples. As shown in Figure 3d and Table S2, the CeO_2_/Ni_2_P/Fe_2_P/NF-3 exhibited the lowest charge transfer resistance (R_ct_) of 0.92 Ω, compared to that of CeO_2_/Ni_2_P/Fe_2_P/NF-1 (2.68 Ω), CeO_2_/Ni_2_P/Fe_2_P/NF-2 (1.29 Ω), CeO_2_/Ni_2_P/Fe_2_P/NF-4 (3.52 Ω), CeO_2_/Ni_2_P/Fe_2_P/NF-5 (3.91 Ω), Ni_2_P/Fe_2_P/NF (6.73 Ω), and bare NF electrode (33.28 Ω). This indicates that the incorporation of CeO_2_ enhances the conductivity and interfacial electron transfer of Ni_2_P/Fe_2_P/NF.

To ensure the long-term performance of the catalyst in the HER, the stability of the catalyst was assessed through chronoamperometry (Figure 3e). The CeO_2_/Ni_2_P/Fe_2_P/NF-3 electrode exhibited exceptional stability, consistently generating hydrogen for up to 200 h at 10 mA cm⁻^2^, with no significant decrease in performance. Based on the aforementioned test results, it is evident that the CeO_2_/Ni_2_P/Fe_2_P/NF-3 catalyst exhibits outstanding catalytic activity and stability in alkaline environments, making it highly valuable for practical applications.

### 3.3. Electrochemical OER Performance

**Figure 4:**
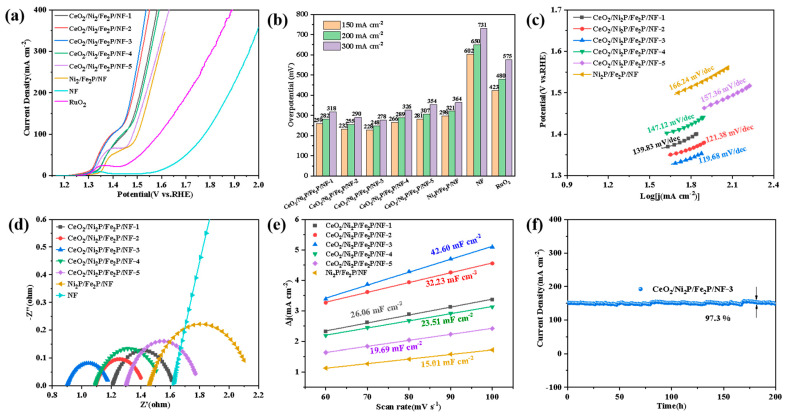
OER electrocatalytic performance in 1.0 M KOH: (**a**) LSV curve, (**b**) overpotential, (**c**) Tafel slope, (**d**) EIS spectrum, (**e**) electric double-layer capacitance values of different samples, and (**f**) durability of CeO_2_/Ni_2_P/Fe_2_P/NF−3 determined by chronoamperometry at 150 mA cm^−2^.

The electrochemical performance of the OER was evaluated in a typical three-electrode system using a 1.0 M KOH electrolyte. Figure 4a displays the LSV curves of CeO_2_/Ni_2_P/Fe_2_P/NF, NF, and RuO_2_. Figure 4b illustrates a comparison of catalytic properties, highlighting that foam nickel, in itself, does not possess any catalytic activity. Among all the comparison samples, the CeO_2_/Ni_2_P/Fe_2_P/NF-3 electrode only requires an overpotential of 228 mV to achieve a current density of 150 mA cm^−2^, which is significantly lower than CeO_2_/Ni_2_P/Fe_2_P/NF-1 (259 mV), CeO_2_/Ni_2_P/Fe_2_P/NF-2 (232 mV), CeO_2_/Ni_2_P/Fe_2_P/NF-4 (266 mV), CeO_2_/Ni_2_P/Fe_2_P/NF-5 (281 mV), and Ni_2_P/Fe_2_P/NF (298 mV) and RuO_2_ (432 mV), highlighting its excellent OER performance. It can be concluded that the addition of Ce content initially enhances the OER activity, followed by a subsequent decline. This phenomenon may be attributed to the fact that small amounts of CeO_2_, as a co-catalyst, are insufficient to effectively promote reaction kinetics. Conversely, excessive CeO_2_ can obscure the active sites of the bimetallic phosphides, thereby hindering their electrochemical performance.

The OER kinetics of the catalysts were assessed using Tafel plots. Figure 4c clearly demonstrates that the Tafel slope of CeO_2_/Ni_2_P/Fe_2_P/NF-3 reaches a minimum value of 119.68 mV dec^−1^, which is lower than that of Ni_2_P/Fe_2_P/NF (166.24 mV dec^−1^) and the other CeO_2_/Ni_2_P/Fe_2_P/NF catalysts with added Ce. This indicates that the electrochemical reaction kinetics of CeO_2_/Ni_2_P/Fe_2_P/NF-3 in the OER are faster, highlighting its improved performance.

To further investigate the reaction kinetics of the catalyst, the EIS of all samples was conducted. As depicted in Figure 4d, the R_ct_ of CeO_2_/Ni_2_P/Fe_2_P/NF-3 is the lowest, indicating excellent conductivity. This can be attributed to the nanosheet array structure’s higher electrolyte infiltration capability, which shortens the electron transfer path and accelerates catalytic kinetics. The introduction of CeO_2_ alters the morphology and electronic structure of CeO_2_/Ni_2_P/Fe_2_P/NF-3, leading to a significant enhancement in charge transport within the active material during the OER process [54,55,56].

Furthermore, to investigate the number of potential active sites and the intrinsic catalytic activity of the catalyst, we conducted CV measurements at a scanning rate of 60–100 mV s^−1^ in the non-faradaic range (see Figure S6). The C_dl_ shown in Figure 4e was obtained from the CV tests. The C_dl_ of CeO_2_/Ni_2_P/Fe_2_P/NF-3 is 42.60 mF cm^−2^, significantly higher than that of CeO_2_/Ni_2_P/Fe_2_P/NF-1 (26.06 mF cm^−2^), CeO_2_/Ni_2_P/Fe_2_P/NF-2 (32.23 mF cm^−2^), CeO_2_/Ni_2_P/Fe_2_P/NF-4 (23.51 mF cm^−2^), CeO_2_/Ni_2_P/Fe_2_P/NF-5 (19.69 mF cm^−2^), and Ni_2_P/Fe_2_P/NF (15.01 mF cm^−2^), indicating that CeO_2_/Ni_2_P/Fe_2_P/NF-3 possesses the largest electrochemically active surface area, exposing more active sites in the reaction [57].

Additionally, a stability test was conducted to assess the stability of the CeO_2_/Ni_2_P/Fe_2_P/NF-3 electrode in OER electrocatalysis. The results indicate that there is negligible attenuation after 200 h of continuous testing at 150 mA cm^−2^, demonstrating its superior OER stability (Figure 4f). This is due to the good conductivity and corrosion resistance of the nickel foam base [58]. Furthermore, the comparable catalysts for the OER and HER are presented in Table 1, respectively.

### 3.4. Overall Water Splitting

**Figure 5:**
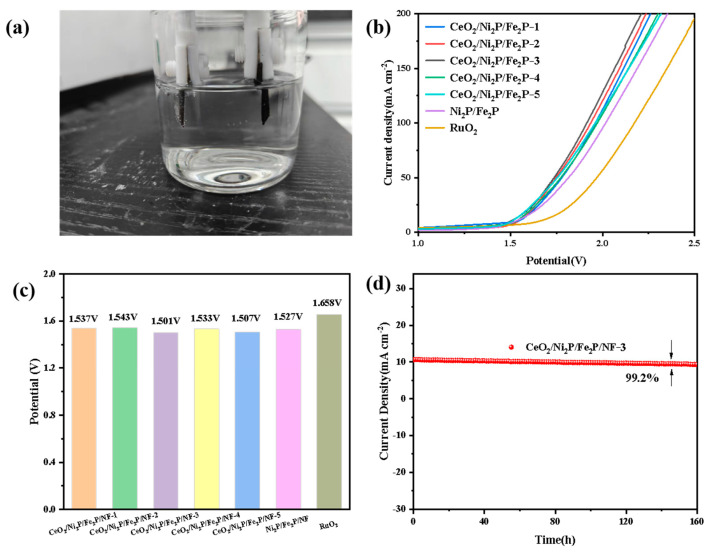
The two-electrode electrolyzers for overall water splitting of CeO_2_/Ni_2_P/Fe_2_P/NF||CeO_2_/Ni_2_P/Fe_2_P/NF, Ni_2_P/Fe_2_P/NF||Ni_2_P/Fe_2_P/NF, and RuO_2_/NF||RuO_2_/NF in 1.0 M KOH. (**a**) The digital photograph of the O_2_ and H_2_ evolution from CeO_2_/Ni_2_P/Fe_2_P/NF; (**b**) polarization curves; (**c**) overpotentials at 10 mA cm^−2^; (**d**) chronopotentiometry test at 10 mA cm^−2^ for CeO_2_/Ni_2_P/Fe_2_P/NF-3||CeO_2_/Ni_2_P/Fe_2_P/NF−3.

To assess the electrocatalytic performance of overall water splitting, we constructed a two-electrode electrolyzer utilizing CeO_2_/Ni_2_P/Fe_2_P/NF-3 as both the anode and cathode in a 1.0 M KOH solution (Figure 5a). The results in Figure 5b,c reveal that the CeO_2_/Ni_2_P/Fe_2_P/NF-3||CeO_2_/Ni_2_P/Fe_2_P/NF-3 electrolyzer achieved a cell voltage of 1.501 V at 10 mA cm^−2^, significantly lower than CeO_2_/Ni_2_P/Fe_2_P/NF-1 (1.537 V), CeO_2_/Ni_2_P/Fe_2_P/NF-2 (1.543 V), CeO_2_/Ni_2_P/Fe_2_P/NF-4 (1.533 V), CeO_2_/Ni_2_P/Fe_2_P/NF-5 (1.507 V), and Ni_2_P/Fe_2_P/NF (1.527 V) and even below RuO_2_ (1.658 V). Furthermore, as shown in Figure 5d, the CeO_2_/Ni_2_P/Fe_2_P/NF-3||CeO_2_/Ni_2_P/Fe_2_P/NF-3 electrode retained 99.2% of its initial activity after 160 h of operation at 10 mA cm^−2^, confirming the excellent durability of the composite in alkaline electrolytes for overall water splitting. Additionally, the cell voltage of the CeO_2_/Ni_2_P/Fe_2_P/NF-3 electrolyzer was comparable to that of other recent related catalysts, as detailed in Table 1.

## 4. Conclusions

In this paper, CeO_2_/Ni_2_P/Fe_2_P/NF nanosheet arrays were synthesized on the surface of nickel foam through hydrothermal and calcination methods. By varying the Ce/(Ni, Fe) molar ratio, a series of CeO_2_/Ni_2_P/Fe_2_P/NF catalysts with different cerium source additions were obtained and utilized as bifunctional catalysts for testing and analysis. The catalyst exhibits superior bifunctional performance, achieving low overpotentials of η_10_ = 87 mV (HER) and η_150_ = 228 mV (OER) with excellent stability, maintaining > 97% current retention after 160 h at 150 mA cm^−2^. This represents a significant improvement over conventional noble metal-based catalysts. The excellent electrocatalytic performance of CeO_2_/Ni_2_P/Fe_2_P/NF-3 is attributed to three synergistic factors: (1) The nanosheet structure provides a large electrochemical active surface area and exposes abundant active sites. (2) Nickel foam substrate ensures super conductivity for fast electron transfer. (3) The well-dispersed CeO_2_ nanoparticles modulate the electronic structure of the bimetallic Ni-Fe center and optimize the adsorption energy of HER and OER intermediates.

## Figures and Tables

**Table 1:** Comparison of water splitting performance between CeO_2_/Ni_2_P/Fe_2_P and other recently reported electrocatalysts in both 1 M KOH.

Electrocatalysts	Overpotential (mV)/HER	Overpotential (mV)/OER	Overall Voltage (V)	Reference
CeO_2_/Ni_2_P/Fe_2_P	87@10 mA cm^−2^	228@150 mA cm^−2^	1.501@10 mA cm^−2^	This work
Ni_3_(NO_3_)_2_(OH)_4_/CeO_2_/NF	120@10 mA cm^−2^	330@50 mA cm^−2^	1.64@10 mA cm^−2^	[56]
NiCo_2_S_4_/Ni_2_P/Fe_2_P/NF	205@50 mA cm^−2^	293@100 mA cm^−2^	1.56@10 mA cm^−2^	[57]
Ni_2_P/Fe_2_P@NiP@NF	105@10 mA cm^−2^	252@100 mA cm^−2^	1.57@10 mA cm^−2^	[58]
Ce@NiCo-LDH	134@50 mA cm^−2^	250@50 mA cm^−2^	1.68@10 mA cm^−2^	[59]
CeFeCoP/NF	97@10 mA cm^−2^	298@50 mA cm^−2^	1.55@10 mA cm^−2^	[60]
NiFe-LDH/MoS_2_–Ni_3_S_2_/NF	79@10 mA cm^−2^	220@50 mA cm^−2^	1.50@10 mA cm^−2^	[61]
Ni/Ni-N_0.28_/NF	63@10 mA cm^−2^	320@20 mA cm^−2^	/	[62]
PNi_3_S_2_/NF	137@10 mA cm^−2^	306@100 mA cm^−2^	1.47@10 mA cm^−2^	[63]

## Data Availability

The original contributions presented in this study are included in the article/Supplementary Materials. Further inquiries can be directed to the corresponding authors.

## Supplementary Materials

The following supporting information can be downloaded at: https://www.mdpi.com/article/10.3390/ma18102221/s1, Figure S1: XRD patterns of CeO_2_/NiFe-LDH/NF. Figure S2. XRD patterns of different catalysts. Figure S3. The SEM images: (a) NiFe-LDH/NF, (b) NiFe-LDH/NF after calcination, (c) Ni_2_P/Fe_2_P/NF, (d) CeO_2_-NiFe-LDH/NF, (e) CeO_2_-NiFe-LDH/NF after calcination, and (f) CeO_2_/Ni_2_P/Fe_2_P-3. Figure S4. The CeO_2_/Ni_2_P/Fe_2_P/NF-3 element-mappings image. Figure S5. XPS spectra of the Ni_2_P/Fe_2_P/NF-3: (a) a full scan survey, (b) P 2p, (c) O 1s. Figure S6. Cyclic voltammograms (CVs) from 60 to 100 mV/s for (a) CeO_2_/Ni_2_P/Fe_2_P/NF-1, (b) CeO_2_/Ni_2_P/Fe_2_P/NF-2, (c) CeO_2_/Ni_2_P/Fe_2_P/NF-3, (d) CeO_2_/Ni_2_P/Fe_2_P/NF-4, (e) CeO_2_/Ni_2_P/Fe_2_P/NF-5, (f) Ni_2_P/Fe_2_P/NF. Table S1. The total element distribution spectrum of CeO_2_/Ni_2_P/Fe_2_P/NF-3. Table S2. Fitting data for various catalysts.
