# Supplementary material for: CeO2-Modified Ni2P/Fe2P as Efficient Bifunctional Electrocatalyst for Water Splitting
Source: Materials (Basel). 2025 May 11;18(10):2221. doi: 10.3390/ma18102221 (PMC12113372; doi:10.3390/ma18102221)
Supplement: Supplementary file 1 [file materials-18-02221-s001.zip › materials-3557045-supplementary.pdf]

Electronic Supplementary Information (ESI)

CeO_2-Modified Ni_2P/Fe_2P as Efficient Bifunctional Electrocatalyst for Water Splitting

**Xinyang Wu [1,2], Dandan Wang [1,2], Yongpeng Ren [1,2,3], Haiwen Zhang [4], Shengyu Yin [4], Ming Yan [4], Yaru Li [1,2,*]
and Shizhong Wei [1,2,3,*]**

[1] School of Materials Science and Engineering, Henan University of Science and Technology,
Luoyang 471000, China; 13243386137@163.com (X.W.); 13343791260@163.com (D.W.);
ren_yp123@163.com (Y.R.)

[2] Henan Key Laboratory of High-Temperature Metal Structural and Functional Materials, National Joint Engineering Research Center for Abrasion Control and Molding of Metal Materials, Henan University of Science and Technology, Luoyang 471000, China

[3] Longmen Laboratory, Luoyang 471000, China

[4] Longbai Group Co., Ltd., Jiaozuo 454191, China; zhanghaiwen@lomonbillions.com (H.Z.); yinshengyu@lomonbillions.com (S.Y.); ym@lomonbillions.com (M.Y.)

* Correspondence: jiayouli138@163.com (Y.L.); wsz@haust.edu.cn (S.W.)

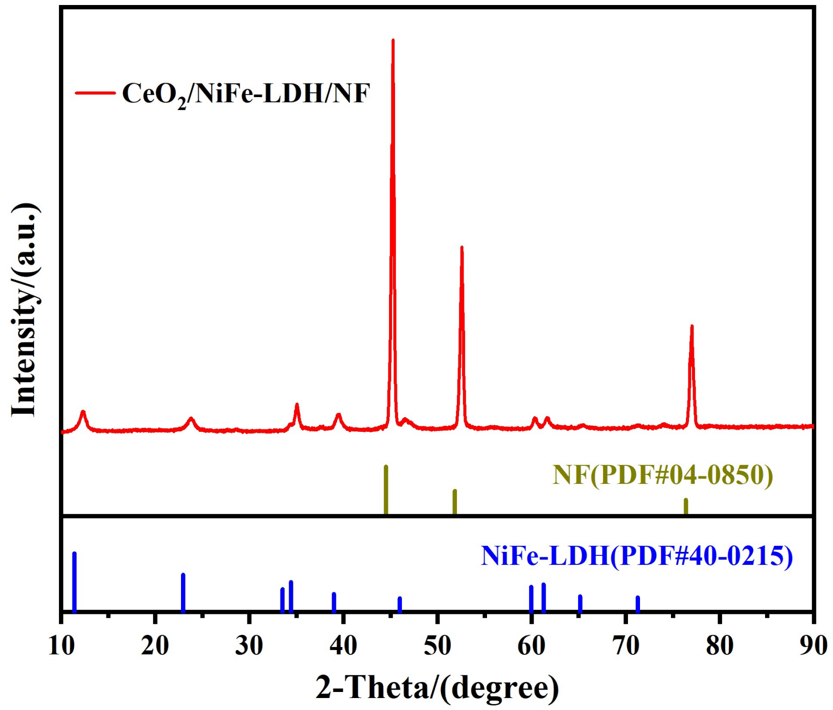

Figure S1. XRD patterns of CeO_2/NiFe-LDH/NF

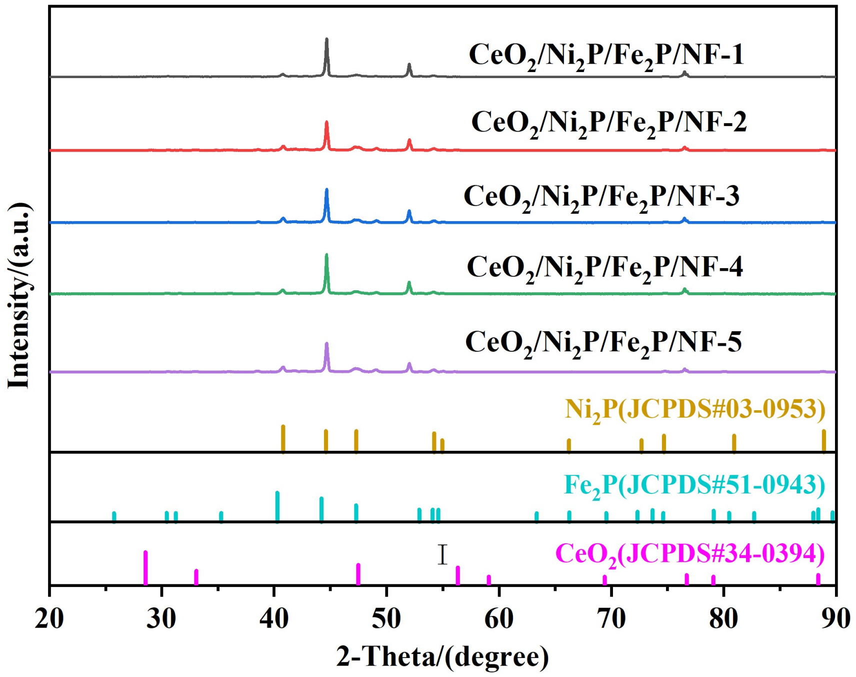

Figure S2. XRD patterns of different catalysts

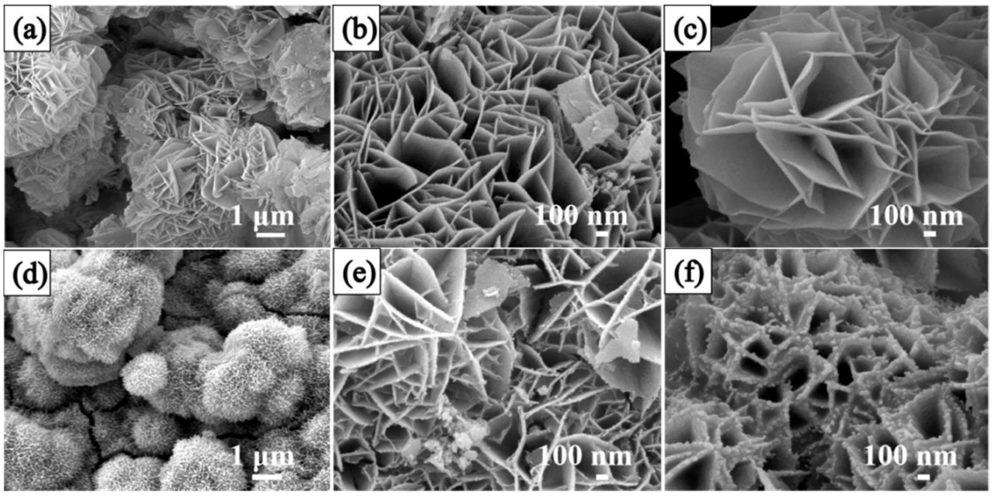

Figure S3. The SEM images: (a) NiFe-LDH/NF, (b) NiFe-LDH/NF after calcination, (c) Ni_2P/Fe_2P/NF, (d) CeO_2-NiFe-LDH/NF, (e) CeO_2-NiFe-LDH/NF after calcination, and (f) $CeO_2/Ni_2P/Fe_2P$-3.

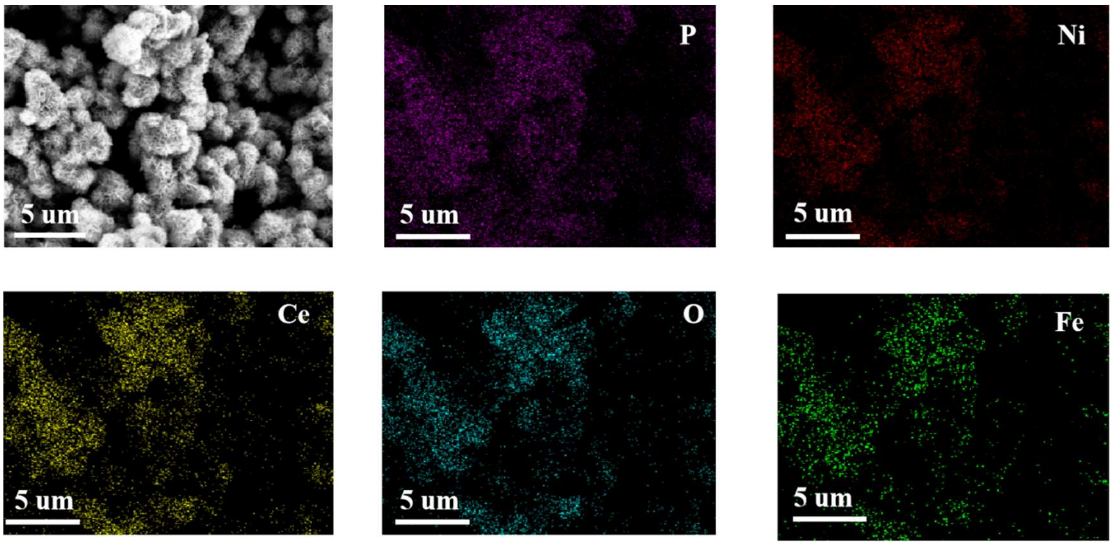

Figure S4. The $CeO_2/Ni_2P/Fe_2P$/NF-3 element-mappings image

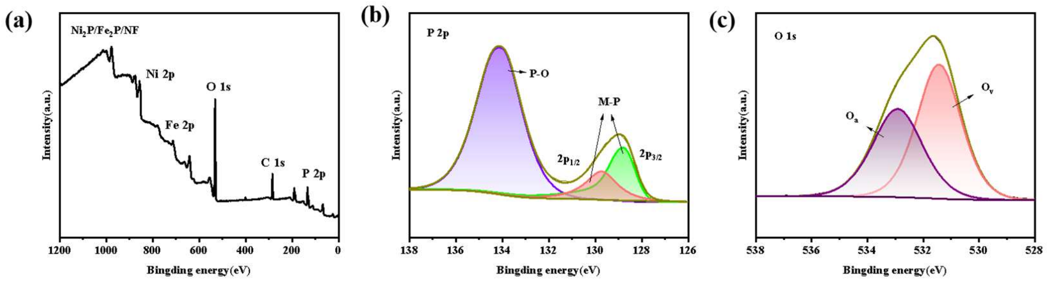

Figure S5. XPS spectra of the Ni_2P/Fe_2P/NF-3: (a) a full scan survey, (b) P 2p, (c) O 1s.

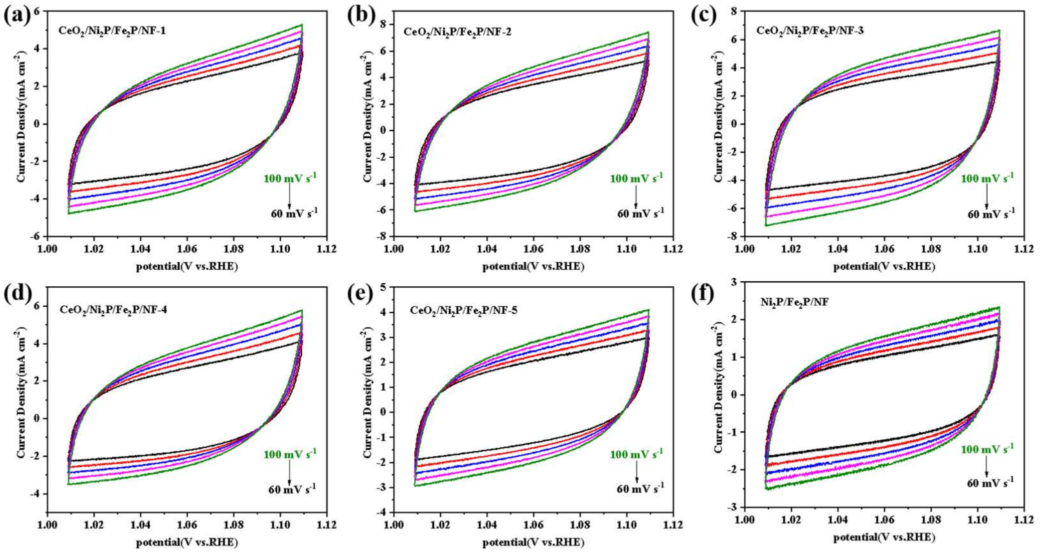

Figure S6. Cyclic voltammograms (CVs) from 60 to 100 mV/s for (a) $CeO_2/Ni_2P/Fe_2P$/NF-1,(b) $CeO_2/Ni_2P/Fe_2P$/NF-2, (c) $CeO_2/Ni_2P/Fe_2P$/NF-3,(d) $CeO_2/Ni_2P/Fe_2P$/NF-4, (e) $CeO_2/Ni_2P/Fe_2P$/ NF-5, (f) Ni_2P/Fe_2P/NF

Table S1. The total element distribution spectrum of $CeO_2/Ni_2P/Fe_2P$/NF-3

**Total element distribution spectrum**	
Element	Wt%
O	5.47
P	17.02
Fe	6.24
Ni	70.84
Ce	0.44
Total amount:	100.00

Table S2. Fitting data for various catalysts

Catalyst	$CeO_2/Ni_2P/Fe_2P$-1	$CeO_2/Ni_2P/Fe_2P$-2	$CeO_2/Ni_2P/Fe_2P$-3	$CeO_2/Ni_2P/Fe_2P$-4	$CeO_2/Ni_2P/Fe_2P$-5	NF	Ni_2P/Fe_2P
R_s	1.12	1.11	1.04	1.12	1.25	1.38	1.25
R_{ct}	2.68	1.29	0.92	3.52	3.91	33.28	6.73
CPE-T	0.17	0.21	0.18	0.08	0.07	0.001	0.11
CPE-P	0.76	0.79	0.74	0.77	0.74	0.85	0.77
